# Bioaccessibility and Risk Assessment of Trace Elements in Mealworms Using Continuous On-Line Leaching Coupled with Inductively Coupled Plasma Mass Spectrometry

**DOI:** 10.3390/foods15091556

**Published:** 2026-04-30

**Authors:** Qiqi Zhang, Ellen Mcgivern, Diane Beauchemin

**Affiliations:** Department of Chemistry, Queen’s University, 90 Bader Lane, Kingston, ON K7L 3N6, Canada

**Keywords:** continuous on-line leaching method, mealworm, inductively coupled plasma mass spectrometry, bioaccessibility, risk assessment

## Abstract

Mealworm (*Tenebrio molitor*) is considered a sustainable protein source and classified as a non-novel food by Health Canada. However, data on safe consumption levels based on bioaccessible metal(loid) concentrations are limited. In this study, a modified continuous on-line leaching method (COLM) coupled with inductively coupled plasma mass spectrometry (ICPMS) was developed to quantify bioaccessible Cr, As, Se, and Cd in mealworm powder. Samples were packed into a transparent polypropylene flash column and sequentially leached with artificial saliva and gastric juice at 37 °C to simulate gastrointestinal digestion, with continuous monitoring of released elements by ICPMS. The proposed method required approximately 70 min per sample as opposed to over 2 h with conventional batch methods. Whereas the bioaccessible concentration of Se was negligible, 13 ± 5 µg/kg Cr, 18 ± 5 µg/kg As and 18 ± 6 µg/kg Cd were released, representing 14%, 15%, and 26% of their total concentration, respectively. Mass balance was verified for Cr, As, Se, and Cd, demonstrating the reliability of the method. Additionally, different sources of elements were revealed by plotting the temporal profile of one element versus that of another element for each gastro-intestinal fluid. A preliminary quantitative risk assessment indicated that adults can safely consume 105 g mealworm per day. Although no significant noncarcinogenic risk was identified, the incremental lifetime cancer risk of 5.2 × 10^−6^ for As and 1 × 10^−6^ for Cr exceeds or equals the Ontario threshold, indicating potential concern. This study is the first to apply the COLM to mealworm while integrating bioaccessibility data for a more realistic risk assessment. However, back-pressure issues result in a relative standard deviation up to 39%.

## 1. Introduction

The rapid growth of the global population and increasing environmental pressure have raised concerns about the sustainability of current food production systems [1]. Conventional livestock production requires large amounts of feed, water, and land, and contributes significantly to greenhouse gas emissions [2,3]. Therefore, alternative and sustainable protein sources are urgently needed. In many countries in Asia, Africa, and Latin America, insects have been consumed for centuries as part of traditional diets [4]. In recent years, edible insects have gained increasing attention because of their high nutritional value and relatively low environmental impact, as they require much less water and land than conventional livestock while emitting less greenhouse gases [4].

Among different edible insect species, mealworms (*Tenebrio molitor*) are one of the most widely studied and commercially produced insects for human consumption. Mealworms contain relatively high protein levels and have a well-balanced amino acid profile, which includes the essential amino acids that can only be obtained from food substances [5]. They also provide beneficial lipids rich in polyunsaturated fatty acids, as well as essential micronutrients, including vitamins (such as vitamin B12), and minerals including Zn and Mg [6]. In fact, their nutritional composition is considered comparable to that of conventional meat [6]. Notably, mealworms exhibit relatively high protein and fat contents compared to conventional meats (Table 1).

In addition to their nutritional value, mealworms show several environmental advantages. Compared with beef, pork, and chicken, mealworms have a more efficient feed conversion ratio and produce lower greenhouse gas emissions [5,7,8,9]. Their production also requires less water and land resources [7,8,9]. Mealworms can be reared on organic by-products, which further improves production sustainability [5]. Large-scale rearing technology has already been established, allowing industrial production under controlled conditions [10]. They can be raised in vertically stacked trays in automated indoor systems [10]. Unlike crickets or flies, mealworms do not hop or fly, which makes farming and handling easier.

Mealworms are not only used for human food but also as animal feed. They have been proposed as a partial replacement for fish meal and soybean meal in feed formulations [6]. In Europe, mealworms have been approved for human consumption and are available as whole dried insects or in powdered form. They are incorporated into products such as pasta, biscuits, and other cereal-based foods [11,12,13]. The flavor of mealworms is generally described as nutty, with mild umami and cereal-like notes [14]. Consumer studies have shown that people are more willing to try mealworms and crickets compared with black soldier fly larvae [15], indicating a relatively higher acceptance potential.

Several studies have evaluated the suitability of mealworms for human consumption when reared on plant-based substrates [16,17]. The use of clean and controlled feed substrates can improve their nutritional quality and safety, supporting their application as food ingredients. Despite these advantages, safety concerns remain. Insects can accumulate hazardous substances from their feed and rearing environment. Potentially toxic elements (PTEs), such as As, were reported to have bioaccumulation factors greater than 1 in mealworms reared on contaminated feed; and the reported As concentration exceeded the current European Commission limit for feed materials (2 mg/kg in complete feed) [18]. Therefore, systematic evaluation of chemical contaminants is necessary to ensure consumer safety.

In Canada, edible insects are regulated under the same legislative framework as conventional foods [19,20]. Depending on their history of consumption, insect products may require novel food assessment by Health Canada [19,20]. Mealworms are classified as non-novel foods [21]. They can be marketed without additional restrictions but must comply with the Safe Food for Canadians Regulations (SFCR) [21,22]. Quantitative risk assessment is therefore essential to support regulatory decision-making and safe consumption guidelines.

**Figure 1:**
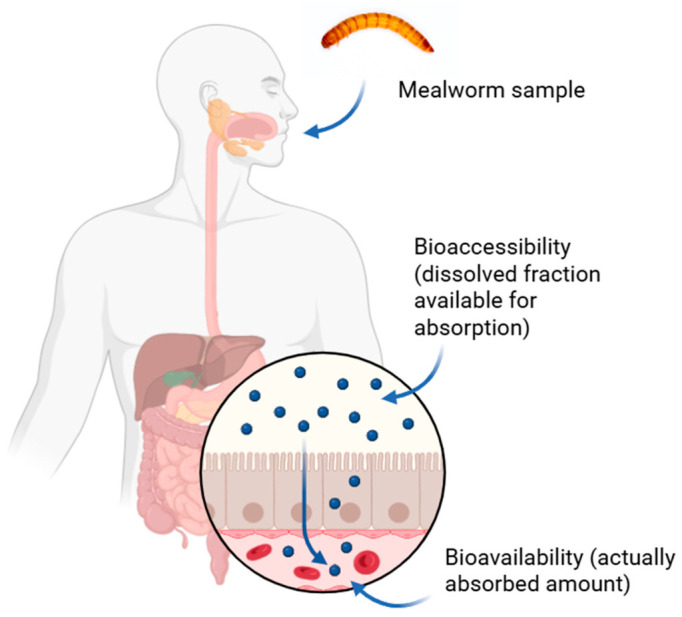
Schematic representation of bioaccessibility and bioavailability in the gastrointestinal tract.

Most previous studies have focused on total concentrations of PTEs in edible insects. However, total concentration does not reflect the fraction that can exert toxic effects. Figure 1 shows that bioaccessibility represents the portion released from the food matrix during digestion and available for absorption into the body. It corresponds to bioavailability (i.e., the portion entering the blood stream, where toxic effects may ensue) in the worst case scenario, and is thus more relevant for estimating actual exposure [23]. There is currently no standardized bioaccessibility method available for food. Some validated in vitro bioaccessibility assays, including the United States (US) Environmental Protection Agency (EPA) method (validated for As and Pb) [24], and unified Bioaccessibility Research Group of Europe method (validated for As, Cd, and Pb) [25], have standard operating procedures for determining human health risks from exposure to inorganic elements in soil.

In this study, a continuous on-line leaching method (COLM) coupled with inductively coupled plasma mass spectrometry (ICPMS) was, for the first time, applied to determine the bioaccessible concentrations of Cr, As, Se, and Cd in mealworms. These elements were selected because they have been commonly measured in edible insect studies and are relevant for food safety assessment, as insects may accumulate trace elements from their feed substrates [26] (for example, Cd, Pb, Ni, As, Hg, and Se have been previously reported in mealworm larvae and diet studies) [27,28]. The bioaccessibility data were integrated with the Health Canada Preliminary Quantitative Risk Assessment (PQRA) framework to evaluate potential human health risks and estimate a safe consumption level [29]. This study provides new evidence to support food safety assessment of mealworms within the Canadian regulatory context.

## 2. Materials and Methods

### 2.1. Instrumentation

Samples were analyzed using a NexION 2000B ICPMS instrument (PerkinElmer, Waltham, MA, USA) equipped with Ni sampler (PerkinElmer, Waltham, MA, USA) and skimmer cones (PerkinElmer, Waltham, MA, USA), and an Al hyper skimmer cone (PerkinElmer, Waltham, MA, USA). Solutions were introduced into a Burgener T2100 nebulizer (Burgener Research Inc, Mississauga, ON, Canada) and a cyclonic spray chamber (PerkinElmer, Waltham, MA, USA). All analytes were measured in He kinetic energy discrimination (KED) mode to alleviate interference from polyatomic ions, such as ^40^Ar^35^Cl^+^ on monoisotopic ^75^As^+^. Daily tuning and sensitivity checks were done using NexION setup solution (1 µg/L Be, Ce, Fe, In, Li, Mg, and Pb in 1% HNO_3_) (PerkinElmer, Waltham, MA, USA) and NexION KED mode setup solution (10 µg/L Co and 1 µg/L Ce in 1% HCl) (PerkinElmer, Waltham, MA, USA). Data acquisition with the COLM was in time-resolved mode with a 25 ms dwell time whereas bulk analysis of residual samples was done in steady-state mode during 232 s. The detailed operating parameters are summarized in Table 2.

### 2.2. Reagents

Artificial gastrointestinal juices were prepared as described in previous studies, without any modification [30,31,32,33]. Artificial saliva was prepared by placing 6.8 g of KH_2_PO_4_ (ACS grade; Sigma-Aldrich, St. Louis, MO, USA) and 77 mL of 0.2 mol/L NaOH (ACS grade; BioShop, Burlington, ON, Canada) in a 1000-mL volumetric flask and making up volume using doubly deionized water (DDW) with the pH adjusted to 6.5 using 0.2 mol/L NaOH. DDW was obtained from an Arium Pro DI water purification system (Sartorius Stedim Biotech, Göttingen, Germany). Gastric juice was prepared by adding 2.0 g of NaCl (ACS grade; Riedel-de Haën, Seelze, Germany), 3.2 g of pepsin (powder, ≥400 units/mg protein; Sigma-Aldrich, St. Louis, MO, USA), and 7.0 mL of sub-boiled HCl (ACS grade; Fisher Scientific, Ottawa, ON, Canada) into a 1000 mL volumetric flask and diluting to the mark with DDW after adjusting its pH to 1.2 using sub-boiled HCl. Intestinal fluid was not used in this work because it can form precipitates quickly, which may interfere with the leaching process and cause operational problems in the COLM system, and the bioaccessible fraction obtained for the same material by a batch method maximizing extraction via ultrasonication was below the limit of quantification [34]. Sub-boiled HNO_3_ (ACS grade; Anachemia, Mississauga, ON, Canada) and H_2_O_2_ (30% *w*/*w*, for ultratrace analysis; Supelco, Sigma-Aldrich, St. Louis, MO, USA) were used for digestion of the residue left after leaching.

The acids were purified through a DST-1000 sub-boiling distillation system (Savillex, Eden Prairie, MN, USA). During preparation, digestive juices were kept at 37 °C in a water bath, mimicking human body temperature and ensuring that the obtained pH value would not change during on-line leaching. The pH was measured by an Accumet Basic AB15 pH Meter (Fisher Scientific, Waltham, MA, USA). Multi-element standard solutions (containing Cr, As, Se, and Cd) were made from 1000 mg/L mono-element stock solutions (SCP Science, Baie d’Urfé, QC, Canada) in 2% sub-boiled nitric acid. Matrix-matched external calibration solutions were prepared by diluting the multi-elemental stock solution with artificial saliva or gastric juice. The mealworm sample analyzed in this study was a certified reference material (VORM-1) provided by the National Research Council Canada (NRC), Ottawa, ON, Canada. In addition to total certified concentrations, bioaccessible concentrations obtained by a conventional batch method are reported for this material [34,35].

### 2.3. Continuous On-Line Leaching Method

**Figure 2:**
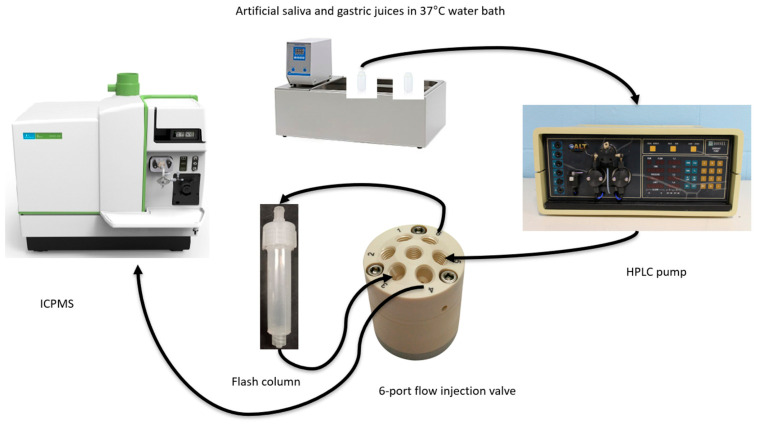
Schematic diagram of the COLM setup, where the arrows indicate the direction of solution flow and the numbers on the valve show the location of its 6 ports.

The COLM setup is illustrated in Figure 2. It was modified from previous work [30,31,32,33,36] to better fit the insect samples that are composed of abundant fats and proteins. An 8 mL empty polyethylene column (iLOK^®^ Empty Solid Load Cartridges; Santai Science Inc, Pointe-Claire, QC, Canada) replaced the previously used stainless steel mini column. This cartridge is 115.1 mm long, with an internal diameter of 12.8 mm, and has a maximum pressure of 200 psi. Mealworm aliquots weighing between 0.1658 and 0.2810 g were sandwiched between two frits. The column was attached to a 6-port flow injection valve (VICI Valco Instruments Co. Inc, Houston, TX, USA). Artificial saliva and gastric juice kept at 37 °C in a HAAKE W19-C10 circulating water bath (Thermo Fisher Scientific (Haake), Karlsruhe, Germany) were sequentially pumped by a Dionex GPM-2 Gradient high-pressure liquid chromatography (HPLC) pump (Thermo Fisher Scientific, Waltham, MA, USA) through the column, which was connected to the nebulizer of the ICPMS instrument. Matrix-matched external calibrations were performed by flow injection using the same setup as in Figure 2 but using a 50-µL sample loop instead of the flash column, with digestive juice used as the carrier solution.

### 2.4. Residue Digestion Method

Each column residue was collected and underwent acid digestion on a hot plate using a modified method from Yang and Sturgeon [37]. Each residue was placed in a pre-cleaned 50 mL polypropylene digestion vessel (Analytichem, Baie d’Urfé, QC, Canada) along with 7 mL sub-boiled HNO_3_ and 0.5 mL H_2_O_2_. The vessels were then sealed and heated at around 115 °C overnight. The resulting digest was finally diluted to 60 mL using DDW. Matrix-matched external calibration standards were prepared to duplicate the acid concentration in the diluted digest.

### 2.5. Mass Balance

Mass balance was performed for quality control and verification of the COLM. The sum of the bioaccessible and residual concentrations was compared to the certified total concentration to see if the values agreed within error.

### 2.6. Data Processing

All data processing was performed using Microsoft Excel 2016 (Microsoft Corporation, Redmond, WA, USA). Analyte peaks obtained during leaching with digestive juices were integrated from the start of leaching until the signal returned to baseline using the mid-point rule. The same method was applied to the transient signal generated by the matrix-matched external calibration. The integrated peak area was then plotted against the injected analyte mass, which was calculated as the injected volume multiplied by the analyte concentration. The calibration equation was applied to sample peak areas to determine the leached analyte mass, which was then divided by the packed column sample mass to obtain analyte concentration in the solid. For the residue analysis, analyte signal was plotted directly as a function of analyte concentration because solutions were continuously nebulized.

The mean of 5 replicates and the corresponding standard deviation were computed. Any outlier identified using Grubbs’ test was excluded prior to calculation of the mean. Comparisons between measured concentrations were performed by first applying an *F*-test to assess if their standard deviations were significantly different, followed by the appropriate Student’s *t*-test at the 95% confidence level.

## 3. Results and Discussion

With growing concerns about the sustainability of global food systems, alternative protein sources such as edible insects are increasingly being considered as a potential solution to future food demand. However, the potential risk related to PTEs contamination needs to be carefully evaluated. Using total elemental concentrations in risk assessment may not accurately reflect actual human health risks, as only the bioavailable fraction (reaching the blood stream), which is greater or equal to bioaccessibility, can exert a toxic effect (Figure 1). Bioaccessibility, which is easier to measure than bioavailability, can thus be used to assess the worst-case scenario: when bioaccessibility equals bioavailability.

To improve the evaluation of PTE exposure from novel protein sources, the COLM was selected over conventional batch methods because it provides dynamic leaching information that cannot be obtained under steady-state conditions. For the first time, a previously developed COLM was applied to mealworms to estimate their safe dosage using a mini-column that was modified to better accommodate samples with high protein and lipid contents, which are typical characteristics of edible insects. Integration of the results obtained in this study with existing federal risk assessment guidelines provides more practical support for food safety evaluation and regulatory assessment.

### 3.1. Flash Column vs. Metal Column

The selection of column material is important for stable COLM performance. When a stainless-steel column was initially used, several limitations were observed. Corrosion resulted from the continuous flushing and frequent cleaning with acidic solutions. In addition, elements originating from the column material, such as Cr and Ni, were detected in the column blanks. Leakage was also frequently observed at the groove, which required additional taping to prevent sample loss.

**Figure 3:**
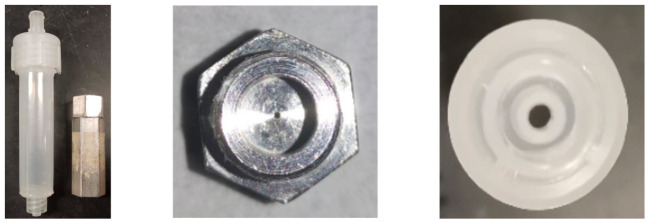
Comparison of flash column and stainless-steel column.

To address these issues, the previously used stainless steel column was replaced by a polyethylene flash column (Figure 3 and Table 3). With the transparent plastic cartridge, the on-line digestion can be visually monitored in real time. The metal-free polyethylene column also results in low metal background in addition to being acid-resistant, reusable, and cost-effective. However, one remaining problem is back pressure as the flash column can only withstand pressure up to 200 psi. Using a column made of polyether ether ketone (PEEK) instead, which can withstand higher pressure, will be tested in future work.

### 3.2. COLM Results and Comparison with Batch Results

**Figure 4:**
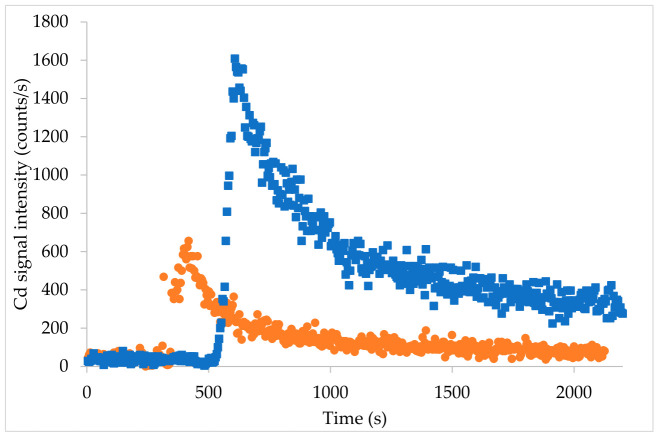
Temporal leaching profiles for ^114^Cd in mealworm by COLM-ICPMS using artificial saliva (orange circles) and gastric fluid (blue squares).

An example of temporal leaching profiles for ^114^Cd with saliva and gastric juice is shown in Figure 4. As the flash column was attached to a flow injection valve, only carrier solution (i.e., digestive juice) first flowed to the nebulizer. When the valve was switched from load mode to injection mode, the HPLC pump pushed digestive fluid through the mini column of sample, releasing bioaccessible analyte.

Pressure fluctuation was observed during leaching with the gastric phase for all replicates. The signal did not fully return to the original carrier baseline, even after 30 min of leaching (Figure 4), which may be indicative of back pressure. For consistency across replicates, the peak was integrated starting from the carrier baseline and ending at the point where the signal change becomes minimal.

Table 4 compares the bioaccessible and residual concentrations measured by the COLM with previous results by a batch method that involved manually homogenizing approximately 0.5 g of sample with 5 mL of artificial saliva solution to ensure wetting of the sample, sonication at 37 °C for 10 min, followed by centrifugation at 4000 rpm for 15 min to collect the supernatant, with the residue subjected to sequential extraction with gastric and intestinal solutions at 37 °C, with manual mixing and sonication (2 h for the gastric phase), followed by centrifugation after each step to obtain the corresponding supernatants [34]. When several isotopes of an element (Cr, Se, Cd) were monitored, the results were consistent between isotopes. Those for ^52^Cr, ^78^Se, and ^114^Cd are summarized in Table 4. While the sum of the analyte concentrations is comparable between methods, the phase distribution is different: except for Cr, a larger fraction of analytes remained in the residue using the COLM than with the independent batch method. This is contrast to previous COLM studies with other food types when comparing the COLM to a batch method involving wrist-action shaking for 10 min with saliva and 2 h with gastric juice for mixing [30,32,36,38]. Indeed, by continuously exposing the sample to fresh reagent, the dissolution equilibrium should be shifted to the right with the COLM, maximizing the amount of analyte released. However, as mentioned above, the batch method in the previous study [34] involved ultrasonication for 10 min with saliva and 2 h with gastric juice for mixing, which is unlikely to be representative of how mixing occurs in the human body. This is supported by a study on seaweed, where 77% Cd and 63% Zn extraction resulted with ultrasonication versus only 26% Cd and 9% Zn extraction with wrist-action shaking using the same gastro-intestinal reagents [33]. Whereas the COLM provides a more physiologically relevant simulation compared to conventional batch extraction methods, it remains an in vitro approximation of human gastrointestinal conditions. For instance, it does not include gut microbiota that may affect the transformation and bioaccessibility of metal species [39]. This limitation should be considered when interpreting the results. As no in vivo data were available to compare to, future work is needed to further validate the performance of the model.

In previous studies on other food types, COLM leaching was done in 5–10 min with saliva and 15–20 min in gastric juice [30,32,36,38] whereas the flash column requires more than double the amount of time. This may indicate that the insect matrix is more resistant to extraction, which will be checked in future work by applying this COLM setup to other food types. Nonetheless, in the case of mealworm, this version of the COLM is only slightly faster (~70 min per sample vs. >2 h for the batch method for the same two leaching reagents). However, it is more dynamic and less susceptible to contamination because leaching is done in a closed system, while offering real-time data, making it suitable for preliminary testing and accurate risk assessment.

### 3.3. Mass Balance Verification

**Figure 5:**
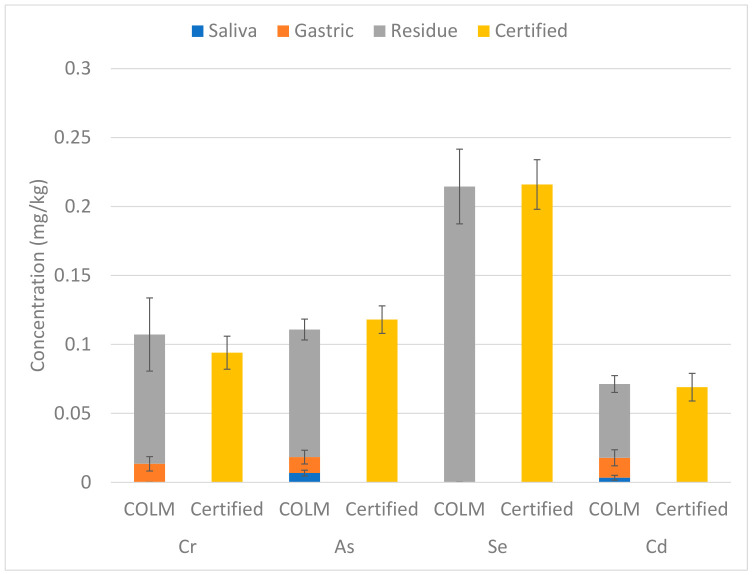
Comparison of the sum of the bioaccessible concentration and the fractions left in the residue phase (mean ± standard deviation, n = 5) with the certified total concentration (mean ± 95% confidence interval).

In any case, the total amounts of Cr, As, Se, and Cd released into the saliva and gastric phases, together with the concentrations remaining in the residual fraction, were compared with the certified total concentrations to evaluate mass balance and validate the analytical method. The combined bioaccessible fractions and residual concentrations were in good agreement with the certified total concentrations (Figure 5), as confirmed by a Student’s *t*-test at the 95% confidence level. To our knowledge, this is the first time that the COLM has been applied to mealworm samples. The results demonstrate that the method is reliable and suitable for the analysis of a complex, high-protein biological matrix. In addition, this work suggests that the COLM could also be extended to other edible insect species for future bioaccessibility and risk assessment studies.

### 3.4. Source Discrimination

**Figure 6:**
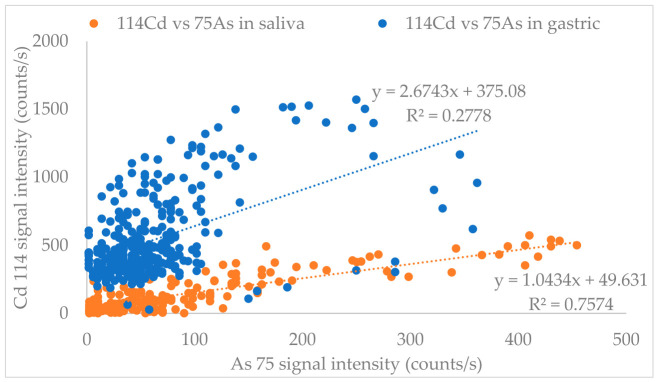
Correlations between the temporal profiles of Cd and As upon leaching with saliva and gastric juice.

Source discrimination is possible using the COLM because analyte leaching kinetics in each digestive phase are observed in real time. The signals of elements originating from the same source tend to correlate with each other [38]. This is shown in Figure 6, where the temporal profiles of ^114^Cd and ^75^As were plotted against each other for the saliva and gastric juice extractions. There is a somewhat linear relationship with saliva, but this is not the case with gastric juice. This may indicate that a common source of As and Cd was dissolved in saliva, but As and Cd were released from different sources with gastric juice. In contrast, conventional batch extraction methods measure only equilibrium concentrations and therefore cannot resolve multiple elemental sources.

### 3.5. Risk Characterization

Risk assessment was performed following Health Canada’s guidance for the Federal Contaminated Site Action Plan (FCSAP) Part I (Federal Contaminated Site Risk Assessment in Canada, Part I: Guidance on Human Health Preliminary Quantitative Risk Assessment, Version 2.0) [29]. A PQRA is a preliminary study and normally contains limited information about PTEs. The PQAR specifies methods and assumptions to ensure that exposures and risks are not underestimated [29]. If the examined exposure risks based on this conservative approach are acceptable or even negligible to human health, then the actual situation would barely cause human health risks [29].

To better assess the risks, the bioaccessible concentration will be used. For the sake of simplicity and being conservative, the potential receptors will be limited to adults (≥20 years) only with oral exposure to food ingestion. All the PTEs are first classified based on their carcinogenicity. A substance could cause both carcinogenic and noncarcinogenic risks depending on multiple factors such as the lifetime exposure frequency. Different risks will lead to different calculations.

The dosages were calculated by using equations for Ingestion of Contaminated Foods (Produce, Fish, Game, etc.) [29].

(1) $$Dose\;(mg/kg\;bw/day)=\frac{{[\sum [C}_{Foodi}\times {IR}_{Foodi}\times {RFA}_{Orali}\times D_{i}]]\times D_{4}}{BW\times 365\times LE}$$

where

*C_Foodi_* = concentration of contaminant in food *i* (mg/kg);

*IR_Foodi_* = receptor ingestion rate for food *i* (kg/day);

*RAF_Orali_* = relative absorption factor from the gastrointestinal tract for contaminant *i* (unitless);

*D_i_* = days per year during which consumption of food *i* will occur;

*D*_4_ = total years exposed to site (to be employed for assessment of carcinogens only);

*BW* = body weight (kg);

*LE* = life expectancy (years) (to be employed for assessment of carcinogens only).

Characterization of cancer and non-cancer risks were calculated by using the following equations:

Incremental Lifetime Cancer Risk (ILCR) = Lifetime Average Daily Dose (LADD) (µg/kg bw/d) × Cancer Slope Factor (µg/kg bw/d)^−1^ (2)

(3) $$Hazard\;quotient\;(HQ)\;=\;\frac{Estimated\;Exposure\;(\frac{\mu g}{(kg\;bw)\times day})}{Tolerable\;Daily\;Intake\;(\frac{\mu g}{(kg\;bw)\times day})}$$

To calculate the dosage, the bioaccessible concentration was used for *C_Foodi_* because it indicates the maximum concentration that might be absorbed into the blood stream. For *IR_Foodi_*, given that mealworms are an alternative protein source, the assumption that daily protein intake is solely coming from this mealworm product was made to assess the worst-case scenario. More specifically, how much mealworms people would consume per day depends on the Recommended Dietary Allowance (RDA) values for total protein [40]. RDA refers to the average daily amount of a nutrient that is enough to meet the needs of nearly all (97–98%) healthy people [40]. The RDA value for total protein of adults (≥20 years) is 0.8 g/kg bw/day [40]. Every 100 g of mealworm contain 54 g of protein [35]. Hence, the *IR_Foodi_* can be calculated by applying the adult’s body weight (70.7 kg). *RAF_Orali_* is always considered to be 100% or 1 [29]. In general, human have a life expectancy of 80 years and adults will have 60 years of ingestion exposure to mealworms.

To account for uncertainty in elemental speciation, risk calculations were conducted assuming that elements were present in their most toxic form with carcinogenic potential, i.e., all the As was inorganic and Cr was in the Cr(VI) form. This approach further contributes to a conservative risk estimate. Toxicological reference values (TRVs) were obtained from data published by authority organizations such as Health Canada (HC), US EPA Integrated Risk Information System, and California EPA. They were used to establish the tolerable daily intake (TDI) values used in the calculation of HQ as well as the oral cancer slope factor (SF) used in the calculation of ILCR and are summarized in Table 5.

The risk assessment results based on the TRVs in Table 5 are summarized in Table 6. If adults consume 105 g of mealworms per serving, once per week (52 weeks per year), no non-carcinogenic health effects are expected to occur. However, the ILCR values for Cr and As are equal or slightly above the acceptable level of 1 × 10^−6^ according to the Ontario criteria. This suggests that long-term exposure to As (especially inorganic forms, including As(III), As(V)), and Cr (in particular Cr(VI)), may pose potential cancer risks. The federal guidelines enable the determination of not only the safe consumption threshold of the food, but also the elemental intake per serving.

A worst-case exposure scenario, that the entire daily protein intake was derived solely from mealworm consumption, was assumed in this study due to the lack of published data on consumption rates of edible insects. This assumption is highly conservative, may not reflect realistic dietary patterns, and may overestimate exposure. In addition, the use of the most toxic forms of elements (such as As(III) and As(V) as well as Cr(VI)) may also lead to higher estimated risks. Nonetheless, this approach was intentionally adopted to avoid underestimation of potential risks in this preliminary assessment. Therefore, the derived threshold of 105 g/day should be interpreted as a conservative upper-bound value, and the actual health risks are likely to be lower. Despite this, all HQ values remained below the safety threshold, supporting the robustness of the assessment. However, the relatively high oral cancer slope factor for arsenic (1.8 mg/kg bw/day)^−1^ [41] indicates a high carcinogenic potency, and thus potential risks should still be carefully considered under long-term exposure. In addition, this assessment was limited to the adult population and did not consider other potential receptor groups such as toddlers, children, and teenagers, mainly due to limited exposure data. Future studies should include these populations to provide a more comprehensive risk characterization.

### 3.6. Technical Challenges and Cost Analysis

The modified COLM system in this study shows several technical challenges when applied to complex food matrices such as mealworms. Compared to staple food matrices reported in previous studies [30,32,36,38], the leaching process takes longer, limiting the improvement in efficiency over conventional batch extraction methods and restricting its application in large-scale or industrial screening. In addition, the use of a flash column operating under low-pressure conditions may affect flow stability and potentially influence the leaching efficiency.

From a cost perspective, the flash column and its consumables are generally low cost compared with more complex high-pressure chromatographic systems. As the modified COLM method takes 58% of the time taken by a batch method, it can provide useful preliminary information for sample evaluation and may be suitable for screening purposes, where quick comparison between samples is needed. It also provides real-time data for an understanding of the different leaching behaviors of elements in samples.

## 4. Conclusions

This study evaluated Cr, As, Se, and Cd in mealworm samples using a modified COLM setup where a flash column was used instead of a metal one. This eliminated corrosion problems that were experienced with a metal column. However, back pressure remained an issue. A smaller fraction of analytes was bioaccessible using the COLM than with a previous batch method that however used ultrasonication for 10 min with saliva and 2 h with gastric juice for mixing, which likely overestimates bioaccessibility in the human body. In any case, mass balance was verified. Correlation or not between the temporal leaching profiles of Cd and As indicates that these elements originate from a common source when leached by saliva but different sources when leached by gastric juice. Risk assessment revealed that adults could safely consume 105 g of mealworms per day, once per week. Potential carcinogenic risks associated with inorganic arsenic and chromium(VI) species warrant further investigation. However, no comparison could be made with in vivo data or standardized in vitro models as neither are available yet.

This assessment, which was very conservative by using mealworm as the sole protein source, focused on the general adult population and did not explicitly consider sensitive subpopulations, such as toddlers, children, or teenagers, who may have higher exposure relative to body weight or increased susceptibility. Future work should include more realistic consumption scenarios, elemental speciation data, and different population groups to improve the accuracy of the risk assessment and better assess the potential carcinogenic risk. The COLM will also be applied to other insects as well as to other food types, such as plant-based alternative protein sources, to determine if leaching occurs faster in other samples or if the flash column modifies the leaching dynamics in a way that favors re-adsorption. Replacing the flash column with a PEEK one will also be explored to further improve the COLM.

## Figures and Tables

**Table 1:** Comparison of mealworm to conventional meats in terms of nutritional values (on a fresh matter basis) [5].

	Mealworm (Powdered Larvae)	Beef (Lean)	Chicken	Pork (Lean)
Protein (%)	44.72	22.3	22.8	22.8
Fat (%)	42.48	1.8	0.9	1.2

**Table 2:** ICPMS (NexION 2000B) operating conditions.

Parameter	Value
Ar nebulizer gas flow rate (L/min)	0.94
Ar auxiliary gas flow rate (L/min)	1.2
Ar plasma gas flow rate (L/min)	18.2
Sample uptake rate (mL/min)	1
Sampling position (mm)	0.50
Radio-frequency power (W)	1600
Analog Stage Voltage (V)	−1675
Pulse Stage Voltage (V)	1300
Quadrupole ion deflector Fixed Voltage (V)	−10
Quadrupole rod offset (V)	−12
Cell entrance voltage (V)	−8
Cell exit voltage (V)	−32
Cell rod offset (V)	−15
Axial field voltage (V)	500
He cell gas flow rate (mL/min)	6
Ions monitored	^52^Cr^+^, ^53^Cr^+^, ^75^As^+^, ^76^Se^+^, ^78^Se^+^, ^82^Se^+^, ^111^Cd^+^, ^112^Cd^+^, and ^114^Cd^+^

**Table 3:** Comparison of COLM columns.

	Metal Column	Flash Column
Current difficulties	Leaking, clogging, corrosion	Back pressure
Column material	Stainless steel	Medical polypropylene cartridge and polyethylene frit
Operating pressure	High pressure	0–200 psi
Column volume	~2 mL	8 mL
Cost	CAD 600	<CAD 3
Column diameter	~1.1 cm	~1.3 cm

**Table 4:** Concentration of Cr, As, Se, and Cd (mg/kg) (mean ± standard deviation, *n* = 5) leached out or left in the residue when using the COLM or a batch method [34].

Analyte	Leaching Method	Saliva	Gastric Juice	Intestinal Fluid	Residue	Bioaccessible + Residue
Cr	COLM	negligible	0.013 ± 0.005	n/a ^a^	0.09 ± 0.03	0.11 ± 0.03
	Batch [34]	0.006 ± 0.007	0.008 ± 0.008	ND	0.11 ± 0.06	0.13 ± 0.06
As	COLM	0.007 ± 0.002	0.012 ± 0.005	n/a ^a^	0.093 ± 0.008	0.111 ± 0.009
	Batch [34]	0.04 ± 0.02	0.044 ± 0.009	0.02 ± 0.01	0.03 ± 0.01	0.13 ± 0.03
Se	COLM	negligible	negligible	n/a ^a^	0.21 ± 0.03	0.21 ± 0.03
	Batch [34]	0.007 ± 0.003	0.07 ± 0.03	0.02 ± 0.02	0.16 ± 0.05	0.26 ± 0.06
Cd	COLM	0.003 ± 0.002	0.014 ± 0.006	n/a ^a^	0.053 ± 0.006	0.071 ± 0.009
	Batch [34]	0.006 ± 0.002	0.049 ± 0.006	0.003 ± 0.004	0.019 ± 0.006	0.077 ± 0.009

^a^ Leaching in intestinal phase was not conducted in this study.

**Table 5:** TRVs used for risk assessment.

PTE	TDI (mg/kg bw/Day)	Cancer SF (mg/kg bw/Day)^−1^
Cr	2.2 × 10^−3^ [41]	0.5 [42]
As	6 × 10^−5^ [43]	1.8 [41]
Se	0.0057 [41]	n/a ^a^
Cd	8.0 × 10^−4^ [41]	n/a ^a^

^a^: oral exposure likely does not cause cancer.

**Table 6:** Risk evaluation results for noncarcinogenic and carcinogenic risks.

		Noncarcinogenic Risks		Carcinogenic Risks	
PTE	Bioaccessible Concentration (mg/kg)	Dose (mg/kg bw/Day)	HQ ^a^	Dose (mg/kg bw/Day)	ILCR ^b^
As	0.018 ± 0.005	3.9 × 10^−6^	6 × 10^−2^	2.9 × 10^−6^	**5.2 × 10^−6^** ^c^
Cr	0.013 ± 0.005	2.8 × 10^−6^	1.3 × 10^−3^	2.1 × 10^−6^	**1 × 10^−6^** ^c^
Se	negligible	NR ^d^	NR ^d^	n/a ^e^	n/a ^e^
Cd	0.018 ± 0.006	3.8 × 10^−6^	4.7 × 10^−3^	n/a ^e^	n/a ^e^

^a^ If HQ ≤ 0.2, the noncarcinogenic risk is deemed negligible. ^b^ If ILCR ≤ 1 × 10^−6^ (Ontario criteria), the associated cancer risk is deemed to be essentially negligible. ^c^ Values bolded exceed guideline values. ^d^ NR = not reported due to negligible bioaccessible concentration. ^e^ n/a: oral exposure does not cause cancer.

## Data Availability

The original contributions presented in this study are included in the article. Further inquiries can be directed to the corresponding author.
